# Benzothiazepane‐Based Curcumin Analogs Show Selective Effects on Respiration, Permeability, and Growth of Gut‐Liver (Co‐Culture) Cell Models

**DOI:** 10.1111/cbdd.70406

**Published:** 2026-09-21

**Authors:** Fuhua Li, Julie De Munck, Felien Morlion, Katarina Magdalenic, Andreja Rajkovic, Matthias D'hooghe, John Van Camp, Charlotte Grootaert

**Affiliations:** ^1^ College of Food Science Southwest University Chongqing People's Republic of China; ^2^ Department of Food Technology, Safety and Health, Faculty of Bioscience Engineering Ghent University Ghent Belgium; ^3^ SynBioC Research Group, Department of Green Chemistry and Technology, Faculty of Bioscience Engineering Ghent University Ghent Belgium

**Keywords:** Caco‐2, co‐culture, curcumin, cytotoxicity, HepG2, mitochondria

## Abstract

Curcumin, a natural polyphenol with anti‐cancer potential, faces limitations due to metabolic instability, and non‐specific toxicity. This study focused on four curcumin analogs (FM038, FM044, FM045, FM050) featuring oxobutenylidene‐substituted benzothiazepane cores bearing pyridinyl or furan substituents. We systematically evaluated their selective toxic effect on human colon (Caco‐2) and liver (HepG2) cells. This study reveals that structural modifications critically govern the selective cytotoxicity of curcumin analogs towards proliferating Caco‐2 and HepG2 cells. Treatment with analogs featuring pyridin‐2‐yl substituents (FM045/FM050) resulted in a slightly reduced potency towards Caco‐2 cells compared to curcumin, whereas FM044 (furan‐2‐yl) was non‐toxic and FM038 (pyridin‐3‐yl, methylated core) maintained similar activity. In contrast, direct exposure to proliferating HepG2 cells resulted in less cytotoxicity compared to the Caco‐2 cell line, which points to a cell‐specific mode‐of‐action in growing cells. When combined in a co‐culture, metabolites generated and/or transported by differentiated Caco‐2 cells significantly decreased HepG2 viability at an apical concentration of 15 μM. Efflux analysis of respiration and acidification showed that FM050 induced the strongest mitochondrial dysfunction in Caco‐2 cells, directly driving cytotoxicity, whereas HepG2 toxicity correlated with intrinsic detoxification capacity rather than mitochondrial disruption. A metabolic shift towards glycolysis was observed in both cell lines, indicating energy pathway modulation to a more dysfunctional phenotype. These findings highlight the dual role of structural optimization (e.g., methylation, heterocyclic substituents) for (cancer) cell‐specific bioactivity and colonic metabolism and may guide future development of curcumin‐based chemopreventive agents after in vivo validation and metabolite characterization.

## Graphical abstract

Benzothiazephane‐based curcuminoids are more potent than curcumin to inhibit liver cancer outgrowth in a Caco‐2/HepG2 coculture model, depending on methylation and heterocyclic substituent pattern. This may be the result of altered absorption, metabolism, and mitochondrial function, which are relevant targets for drug design.

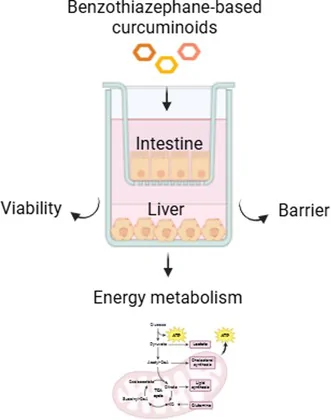

## 1 Introduction

Curcumin, the yellow pigment in turmeric ( *Curcuma longa* ), has garnered significant attention for its anti‐cancer properties, including chemopreventive, anti‐inflammatory, and pro‐apoptotic effects (Kunnumakkara et al. 2017). However, its low aqueous solubility and non‐specificity hinder its efficacy in clinical applications (Ahmed et al. 2025; Habib et al. 2020; Li et al. 2024).

Structural modifications of curcumin have emerged as a strategic approach to enhance pharmacokinetic properties, with studies demonstrating that introducing functional groups—such as heterocyclic moieties or hybrid frameworks—can amplify target specificity and bioactivity (Ahmed et al. 2025). For instance, hybrid molecules combining curcumin with bioactive scaffolds exhibit synergistic effects by modulating multiple signaling pathways, including NF‐κB and mitochondria‐induced apoptosis (Gupta et al. 2011). The natural derivatives of curcumin, demethoxycurcumin and bisdemethoxycurcumin, have higher antiproliferative activity (Mbese et al. 2019; Shi et al. 2021). This underscores the potential of structural optimization to improve therapeutic efficacy of natural curcumin. However, many gaps remain in understanding the specificity or structure–activity relationships (SAR) between the structural features of synthetic curcumin analogs and their functional activities. This poses a challenge for the targeted synthesis of curcumin analogs with desired bioactivity profiles.

Mitochondria play an important role in cellular bioenergetics, amino acid biosynthesis, fatty acid oxidation, and homeostasis of cellular calcium ions (Bock and Tait 2020). In rapidly multiplying cancer cells, mitochondria participate in the proliferation, progression, and development of drug resistance, leading to sensitivity of cancer cells to the disturbance of mitochondrial functions (Abate et al. 2020). Therefore, mitochondria have been an attractive target in the design of tumour‐targeted anti‐cancer therapeutics. Recent advances highlight mitochondrial dysfunction as a pivotal mediator of curcumin‐induced cytotoxicity, yet the metabolic reprogramming effects of curcumin analogs in cancer‐specific contexts are poorly understood (Mortezaee et al. 2019; Waseem and Parvez 2013).

Cell line models have been widely employed to assess the anti‐cancer potential of dietary compounds. Yet, they neglect the interplay between intestinal metabolism and hepatic responses, which critically influences compound bioactivity (Gupta et al. 2011). Curcumin, its synthetic analogs, or extracts rich in curcumin exhibited remarkable cytotoxicity against Caco‐2 cells (Habib et al. 2020; El‐Sherbiny et al. 2022; Sharma et al. 2023) and HepG2 cells (Becit et al. 2020; Cao et al. 2007; Darwesh and Elbialy 2021; Jiang et al. 2013; Yu et al. 2022). However, in such cell‐based studies, compounds are often directly administered to hepatic cells without accounting for metabolic interactions. Biotransformation, such as Phase I and II processes, typically occurs in intestinal and hepatic cells and may lead to profound effects on compound solubility, intestinal and blood transport, tissue distribution, and bioactivity.

This study aims to unravel some mode‐of‐actions related to the potential selective anti‐cancer effects of four novel curcumin analogs (FM038, FM044, FM045, FM050) (Magdalenić et al. 2024) with oxobutenylidene‐substituted benzothiazepane cores and heterocyclic substituents (pyridinyl or furan groups). We systematically evaluate their bioactivity in both monolayers (Caco‐2, HepG2) and co‐culture models (Caco‐2/HepG2), simulating intestinal‐hepatic metabolic interactions. Furthermore, real‐time efflux analysis was applied to elucidate mechanistic links between structural modifications, mitochondrial (dys)function, energy metabolism, and cytotoxicity.

## 2 Materials and Methods

### 2.1 Analog Structure

**FIGURE 1:**
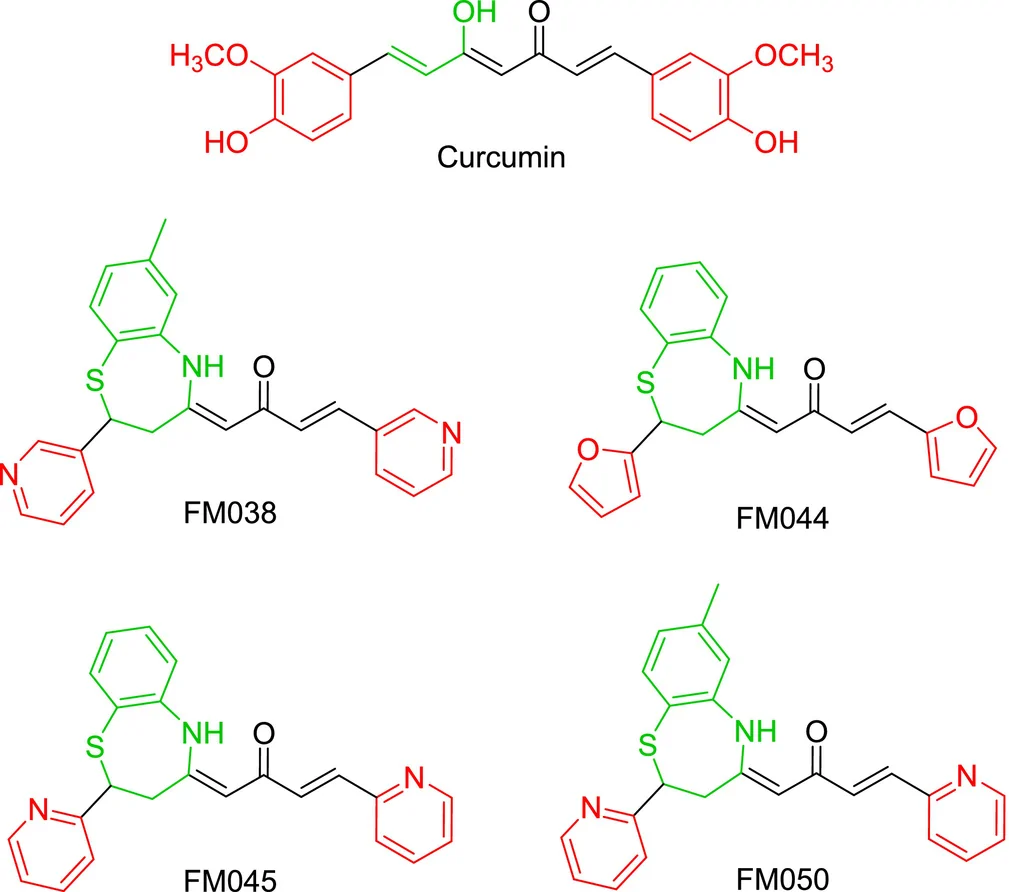
The molecular structure of curcumin and its analogs (FM038, FM044, FM045, and FM050).

Curcumin is a pigment with a diketone structure, composed of two ortho‐methoxylated phenols and a β‐diketone, and contains functional groups such as ether bonds, phenolic hydroxy groups, enolizable ketone carbonyl groups, and carbon–carbon double bonds. The chemical structure of the four curcumin analogs, FM038, FM044, FM045, and FM050, is shown in Figure 1. The chemical structure of FM038 concerns an oxobutenylidene‐substituted methylbenzothiazepane system bearing two pyridin‐3‐yl substituents, and FM044 is characterized by an oxobutenylidene‐substituted benzothiazepane system bearing two furan‐2‐yl substituents. FM045 accommodates an oxobutenylidene‐substituted benzothiazepane system bearing two pyridin‐2‐yl substituents, and structure FM050 consists of an oxobutenylidene‐substituted methylbenzothiazepane system bearing two pyridin‐2‐yl substituents. Accordingly, FM045 and FM050 share identical peripheric pyridin‐2‐yl substituents but differ in the presence of a methyl group on the benzothiazepane core (FM050: methylated). FM044 is functionalized with furan‐2‐yl substituents, while FM038 combines a methylated core with pyridin‐3‐yl groups, highlighting systematic variations in both heterocyclic substituents and core modifications among the derivatives. All analogs were constructed in‐house according to the protocol of Magdalenić et al. (2024), and were stored in a 50 mM stock solution in DMSO at −20°C.

### 2.2 Materials

Corning cell culture flasks (T25 and T75) and plates (96‐well, 24‐well, 24‐Transwell) were purchased from Greiner Bio‐One (Vilvoorde, Belgium). 96‐well Agilent Seahorse XF Cell Culture Microplates and sensor cartridges were purchased from Agilent Technologies Inc. (Ghent, Belgium). DMSO was purchased from Novolab (Geraardsbergen, Belgium). MTT powder and Tris‐buffer were purchased from Merck (Overijse, Belgium). Curcumin (Merck) was stored as a 100 mM stock solution in DMSO at −20°C.

HepG2 cells and Caco‐2 cells were obtained from the American Type Culture Collection (Molsheim, France). All cell culture materials, including Dulbecco's modified Eagle medium (DMEM) (Gibco, GlutaMAX) with 4.5 g/L glucose, pyruvate, penicillin/streptomycin (1%) and non‐essential amino acids (1%), were purchased from Gibco Life Technologies. Fetal bovine serum (added at 10%) was purchased from Avantor (Leuven, Belgium).

Chemicals involved in Seahorse XF Mito Stress Tests including Seahorse XF DMEM, 1 mM pyruvate, 2 mM glutamine, 10 mM glucose, the Seahorse XF Cell Mito Stress Test Kit, and the relevant reagents (oligomycin, FCCP, and a mix of rotenone/antimycin A) were purchased from Agilent Technologies Inc.

### 2.3 Cell Culture

HepG2 and Caco‐2 cell lines were cultured under standard conditions (37°C, 5%–10% CO_2_, 95% relative humidity). Routine monitoring of cell viability and morphology was performed by phase‐contrast microscopy (Zeiss, Zaventem, Belgium), and culture media were refreshed at 2–3‐day intervals. Sub‐culturing by trypsinization was initiated at 80% confluence to preserve phenotypic consistency. For all experiments, cell passage number was restricted to 12–35.

Monocultures of HepG2 and Caco‐2 cells were seeded at 2 × 10^4^ cells per well in 96‐well plates, respectively. For the cytotoxicity assays, the following concentrations were applied for 24 h to calculate the IC50: 0, 0.6, 1, 3, 7.5, 10, 15, 20, 30, and 40 μM. The coculture model was set according to van Laar et al. (2022). Briefly, Caco‐2 cells were seeded in the upper 24‐well inserts at 1.5 × 10^5^ cells per well and were differentiated for 21 days. Caco‐2 barrier integrity was measured via transepithelial electrical resistance (TEER) using an automated REMS system (World Precision Instruments, Sarasota, FL, USA). HepG2 cells were seeded in the basal compartment at 5 × 10^4^ cells per well and incubated for 48 h before coculturing. Differentiated Caco‐2 and HepG2 cells were cocultured for 24 h. Next, the apical side of Caco‐2 monolayers was treated with curcumin or its derivatives (7 or 15 μM) for 24 h, allowing metabolites secreted by Caco‐2 cells to reach HepG2 cells in the basal compartment.

### 2.4 Cytotoxicity

Cytotoxicity was quantified via the tetrazolium salt (MTT) (Merck) assay (Chen et al. 2022). Briefly, 1 day after cell seeding, curcumin and analogs, diluted in DMEM, were added to the cells at different concentrations (200 μL/well). After 24 h incubation, 100 μL/well medium was removed and 20 μL MTT (5 mg/mL in PBS) was added. The plate was incubated for 2 h at 37°C to convert MTT to formazan. Next, media were carefully removed, and formazan crystals were solubilized in 200 μL DMSO by pipetting. Absorbance was measured at 570 nm using a SpectraMax Microplate Reader (Molecular Devices, Berkshire, UK). Results are presented as the percentage of viability (%) relative to the untreated control based on the following formula:

$$\text{Viability}\;\left(\%\right)=100\times \frac{\text{absorbance treated cells}-\text{absorbance DMSO}}{\text{absorbance untreated cells}-\text{absorbance DMSO}}$$

At least three wells per treatment were considered per plate, and each treatment was chosen in the optimal concentration range to calculate the IC_50_ values using the nonlinear regression module of SPSS 27.0 software following its built‐in protocol.

### 2.5 Seahorse Extra‐Cellular Flux Analysis of Mitochondrial Respiration and Glycolysis

Bio‐energetic profiling of Caco‐2 and HepG2 cells was conducted to evaluate metabolic effects induced by curcumin and analogs (7.5 μM) using an Agilent Seahorse XF96 Analyzer. The XF Cell Mito Stress Test was applied to quantify oxygen consumption rate (OCR) and extracellular acidification rate (ECAR) under sequential modulation of electron transport chain (ETC) activity. Cells were seeded at 8000 per well (80 μL) in Seahorse 96‐well microplates and equilibrated under standard culture conditions (37°C, 10% CO_2_) for 24 h. Next, cells were treated with curcuminoids for 24 h. In parallel, the sensor cartridge was hydrated at 37°C overnight.

Prior to flux analysis, cells were washed twice with Seahorse base medium DMEM (+HEPES, pH 7.4) supplemented with 10 mM glucose, 2 mM sodium pyruvate and 2 mM glutamine, and incubated for 1 h at 37°C without CO_2_. Stressors were loaded in the cartridge ports and OCR/ECAR was assessed after sequential injection of 1 μM oligomycin (ATP‐ase inhibitor), 0.25 μM FCCP (membrane uncoupling agent), and 0.5 μM rotenone/antimycin A (electron transport chain shutdown). The program was as follows: for each stressor injection: 3 cycles of 1 min mix, 1 min wait, 2 min OCR/ECAR recording. Bioenergetic indices such as basal respiration, ATP‐coupled respiration, maximal respiratory capacity, and proton leak‐associated OCR were calculated using the Wave software, based on at least three measurements.

### 2.6 Live/Dead Cell Staining

Cell viability staining was performed using the Live‐or‐Dye Fixable Viability Staining Kit according to the manufacturer's protocol. Caco‐2 cells in the coculture setup were treated with curcumin or analogs for 96 h to allow for sufficient transport of the compounds to the HepG2 cells seeded in the basal side to exert an effect. Next, basal medium was removed, and cells were washed twice with PBS before staining with a 1:1000 dilution of Fixable Dead Cell Dye in PBS. After 30 min incubation at room temperature under light‐protected conditions, cells were washed with PBS and fixed with 4% paraformaldehyde for 15 min. After washing twice with PBS, cells were imaged using the IncuCyte Live‐Cell Analysis System. Pictures were analyzed with the IncuCyte ZOOM 2018A Software package.

### 2.7 Data Analysis

Statistical significance was assessed using one‐way ANOVA with Tukey's test (for normally distributed data) or using one‐way ANOVA with Dunn's test (for non‐normally distributed data) (*p* < 0.05) via GraphPad Prism (version 9.5, GraphPad Software, USA). Graphical representations and data visualization were generated using OriginPro 2020 (OriginLab Corporation, MA, USA).

## 3 Results

### 3.1 Cytotoxicity of Curcumin (Analogs) on Cell Monolayers

The cytotoxic effects of curcumin and analogs on Caco‐2 and HepG2 cells were evaluated by MTT (Figures S1 and S2). IC_50_ values are summarized in Table 1.

**TABLE 1:** Cytotoxic effects of curcumin and its analogs on Caco‐2 and HepG2 cells in a mono‐layer model based on an MTT assay.

Compounds	Caco‐2 (IC_50_ μM)	HepG2 (IC_50_ μM)
Curcumin	28.79 ± 0.29^b^	nc
FM038	26.28 ± 0.17^b^	nc
FM044	nc	nc
FM045	36.98 ± 3.27^a^	nc
FM050	33.41 ± 1.92^a^	nc

*Note:* Nc: IC50 values not calculated due to the non‐toxic effect within the tested concentration range (0.6–40 μM). Distinct uppercase and lowercase letters denote significant differences in data within the same column.

The compounds can be ranked from high to low cytotoxicity on Caco‐2 cells in the following order: FM038 (IC_50_: 26.28 μM) and curcumin (IC_50_: 28.79 μM) > FM050 (IC_50_: 33.41 μM) and FM045 (IC_50_: 36.98 μM) > FM044 (IC_50_: > 40 μM) (Table 1). FM044 showed negligible cytotoxicity, suggesting that its structural modifications impair bioactivity. Therefore, it could be inferred that pyridin‐3‐yl substitution (FM038) combined with a methylated benzothiazepane core appears critical for retaining anti‐proliferative potency comparable to curcumin, suggesting that both the positional isomerism of the heterocyclic group (pyridin‐3‐yl vs. pyridin‐2‐yl) and core methylation synergistically enhance bioactivity. In contrast, pyridin‐2‐yl derivatives (FM045, FM050) showed decreased cytotoxicity despite methyl group incorporation in FM050, indicating limited benefits from core methylation when paired with this substitution pattern. The near‐absence of toxicity in FM044, featuring furan‐2‐yl substituents, underscores the importance of nitrogen‐containing heterocycles for cytotoxic efficacy. These trends collectively highlight that (1) aromatic substituent identity (pyridine > furan) and positional isomerism (3‐yl > 2‐yl) dominate activity over core methylation, and (2) methylated cores selectively increase activity when combined with optimal heterocyclic motifs, as evidenced by FM050 versus FM045. Interestingly, whereas these components (excluding FM044) demonstrated varying anti‐proliferative effects against Caco‐2 cells, none showed activity in HepG2 cells at the tested concentrations. For the following longer‐term co‐culture experiment, 7.5 and 15 μM concentrations were chosen because they showed marginal cytotoxicity for growing Caco‐2 cells (Figures S1 and S2).

### 3.2 Impact of Curcumin and Its Analogs on Caco‐2 Cell Monolayer Integrity and HepG2 Toxicity in a Co‐Culture Model

**FIGURE 2:**
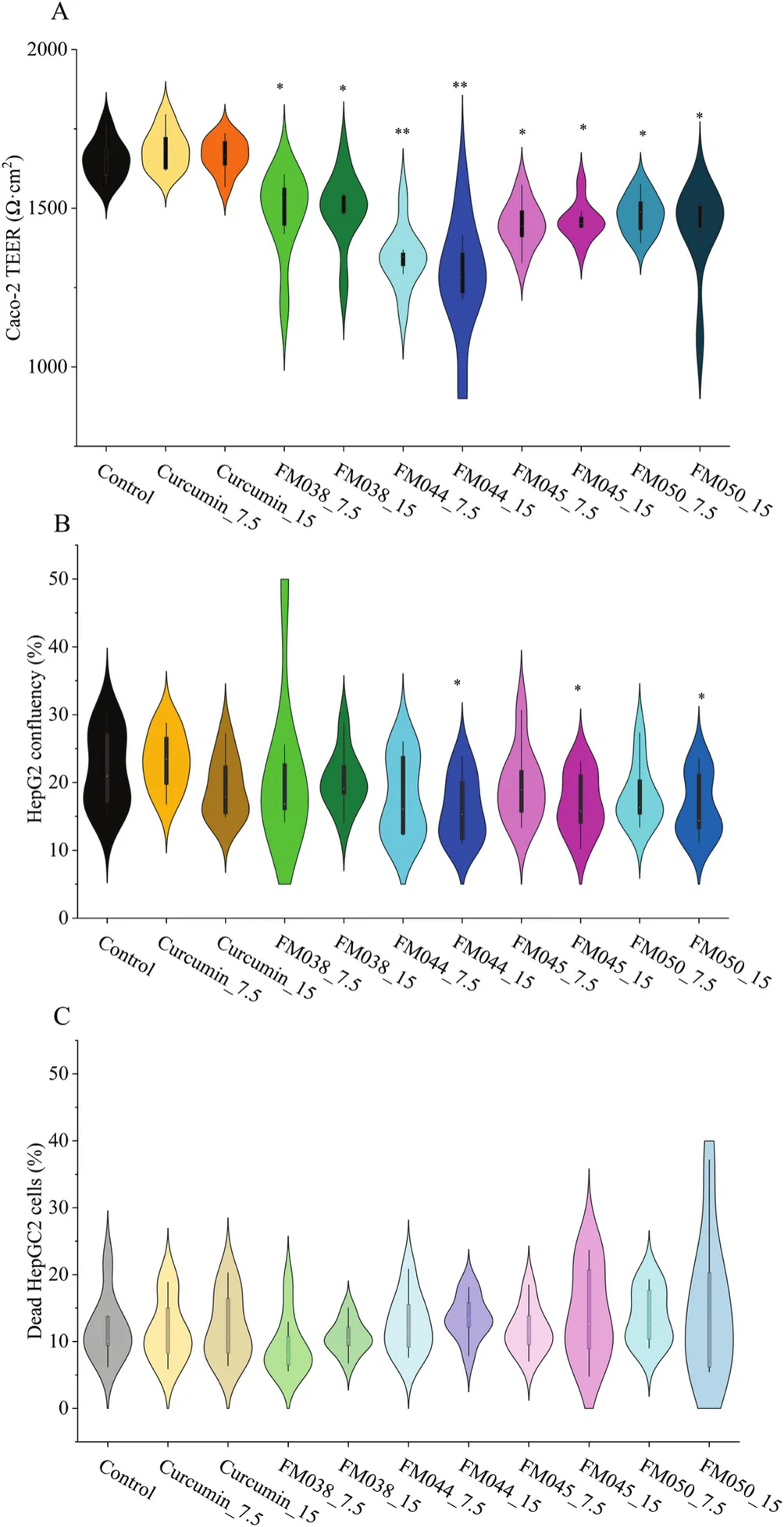
Effect of curcuminoids on the Caco‐2/HepG2 coculture model. TEER values (Ω*cm^2^) of Caco‐2 cells (A), HepG2 cell confluence (%) (B), and dead cell percentage (%) (C) in co‐culture models following apical treatment with curcumin or analogs at concentrations of 7.5 or 15 μM for 96 h. Results are based on 16 repeats (8 plates with duplicate wells each plate) for the treatments, and 32 repeats (8 plates with 4 replicate wells) for the control. Significantly different results compared to the untreated control are indicated with *p < 0.05 or **p < 0.01.

In the co‐culture model, the apical side of the differentiated Caco‐2 monolayer was directly exposed to curcumin or analogs, thereby serving as an intestinal barrier interface. HepG2 cells in the basal compartment were thus exposed to compounds transported by the Caco‐2 cells, their metabolites and/or other factors secreted by Caco‐2 cells as an indirect response to the compound. Figure 2 shows the Caco‐2 TEER values, as well as the HepG2 cell confluence and number of dead cells after 96 h of treatment. These metrics over the entire duration of the experiment are summarized in Figure S3 (Caco‐2) and Figure S4 (HepG2).

Initial TEER values were higher than 500 Ω*cm^2^, indicating a successfully developed barrier (Zeng et al. 2017), although the variability at the start was relatively high (Figure S3). As these measurements were conducted on Day 21 without prior medium refreshment, variability may have been attributed to pH fluctuations, metabolite accumulation, and positional variations across the plate (Vigh et al. 2021). Nevertheless, upon medium refreshment, TEER converged again to higher and considerably less variable values. Upon 96 h of treatment, curcumin (7.5 and 15 μM) showed a similar TEER compared to the control (Figure 2A) throughout the full treatment period (Figure S3). In contrast, the four analogs, and especially FM044, significantly decreased TEER (Figure 2A, *p* < 0.01), but as this decrease was moderate (appr. 20%), we may conclude that the barrier was not destroyed, so the observed impact on HepG2 cells was most probably not due to direct compound diffusion through the Transwell filter.

**FIGURE 3:**
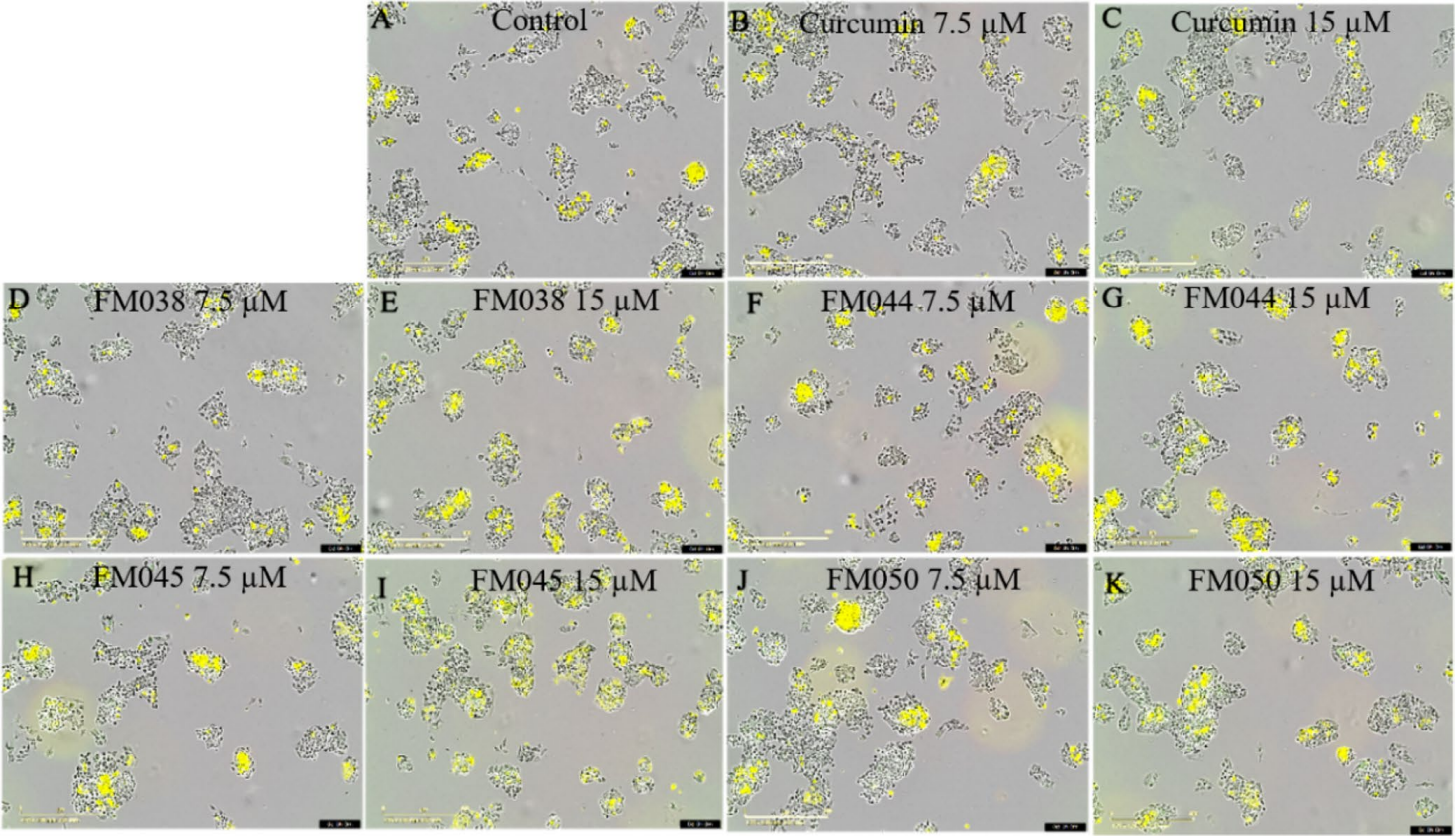
Representative pictures of cell confluency and viability of HepG2 cells after 96 h treatment of the co‐culture model. Dead cells with high intracellular fluorescence are indicated by the yellow color. Untreated cells (A), and cells treated with curcumin at 7.5 μM (B) and 15 μM (C); FM038 at 7.5 μM (D) and 15 μM (E); FM044 at 7.5 μM (F) and 15 μM (G); FM045 at 7.5 μM (H) and 15 μM (I); and FM050 at 7.5 μM (J) and 15 μM (K).

For HepG2 cells located at the basal side, apical treatment of Caco‐2 cells with compound FM044, FM045, and FM050 at 15 μM (and not at 7.5 μM) resulted in significantly decreased cell confluency (Figure 2B, *p* < 0.01), whereas this was not observed for curcumin and FM038. Violin plots of HepG2 cells stained with a live/dead stain (Figure 2C) did not show an overall significant increase in dead cells for all compounds, but the more spread shape of the plot showed that, especially for compounds FM045 and FM050, more wells with a higher number of dead cells were observed. Figure 3 shows that dead cells were mainly localized within cellular aggregates, with denser clusters generally showing darker staining intensities, correlating with increased cell death.

These findings suggest that Caco‐2 cell metabolism may attenuate the cytotoxicity of these compounds. Alternatively, the lower cytotoxicity may also arise from decreased effective concentrations; specifically, the actual concentration reaching HepG2 cells is likely substantially lower than the initial concentration applied to Caco‐2 cells.

### 3.3 Bioenergetic Effects of Curcumin and Analogs

**FIGURE 4:**
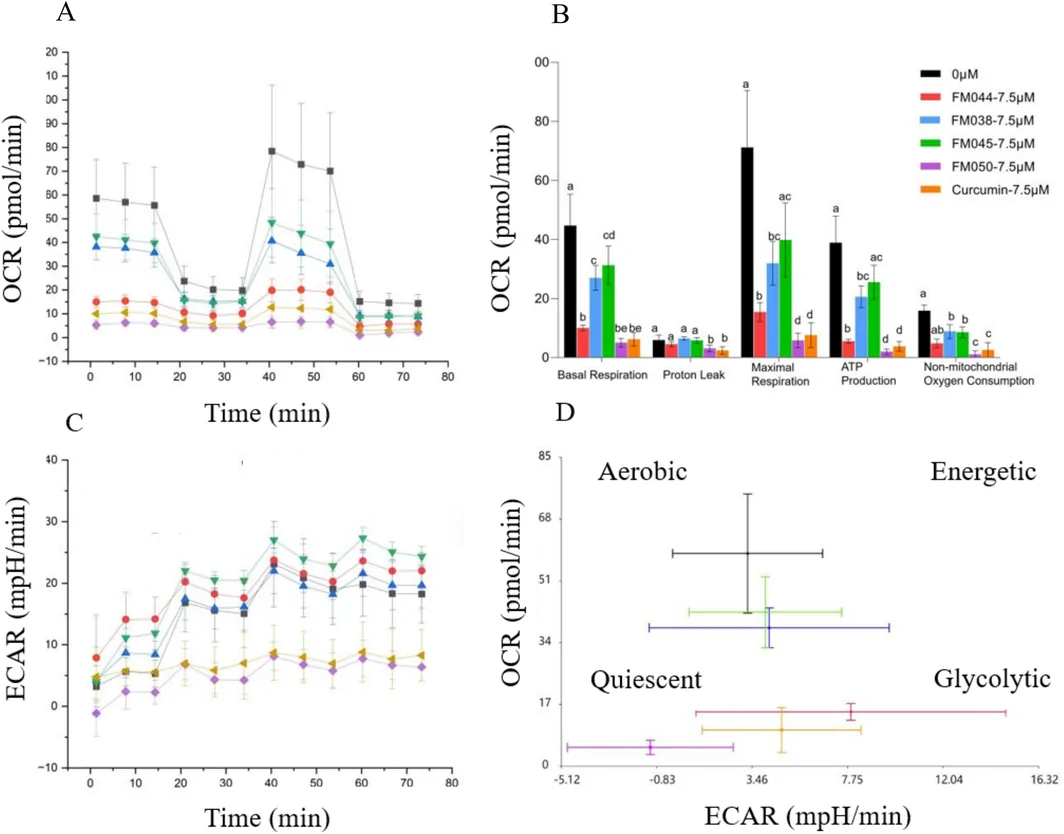
Effect of curcuminoids on energy metabolism in Caco‐2 cells. The Oxygen Consumption Rate (OCR) (A), different mitochondrial parameters (B), Extracellular Acidification Rate (ECAR) (C), and energy map (D) of proliferating Caco‐2 cells treated with curcumin (orange), FM038 (blue), FM044 (red), FM045 (green), FM050 (purple), or untreated (black) at a concentration of 7.5 μM for 24 h. Data (1 plate with at least five replicate wells) are expressed as mean ± standard deviation. Mean values with different letters (a–d) within each mitochondrial parameter indicate significant differences (*p* < 0.05) between different treatments according to one way ANOVA test followed by Tukey's test (for normally distributed data), or Dunn's test (for non‐normally distributed data).

**FIGURE 5:**
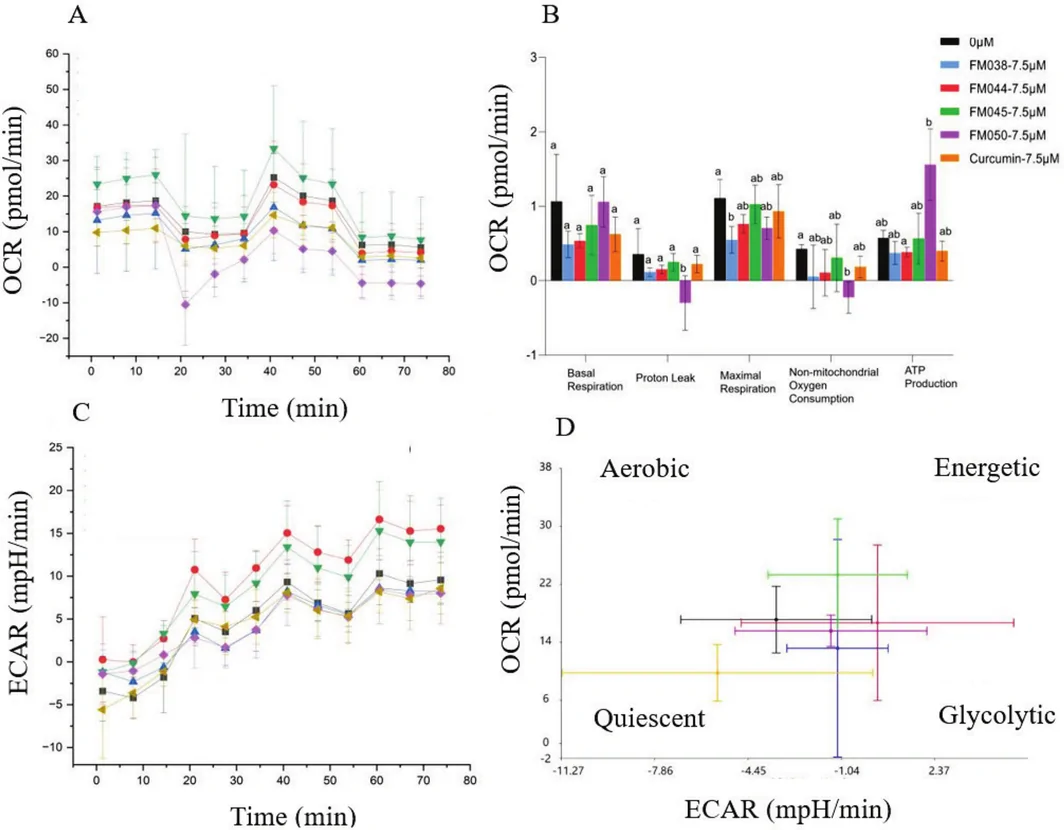
Effect of curcuminoids on energy metabolism in HepG2 cells. The Oxygen Consumption Rate (OCR) (A), different mitochondrial parameters (B), Extracellular Acidification Rate (ECAR) (C), and energy map (D) of proliferating HepG2 cells treated with curcumin (orange), FM038 (blue), FM044 (red), FM045 (green), FM050 (purple), or untreated (black) at a concentration of7.5 μM for 24 h. Data (1 plate with at least five replicate wells) are expressed as mean ± standard deviation. Mean values with different letters (a–d) within each mitochondrial parameter indicate significant differences (*p* < 0.05) between different treatments according to one way ANOVA test followed by Tukey's test (for normally distributed data), or Dunn's test (for non‐normally distributed data).

To test effects on cell bioenergetics, concentrations lower than 15 μM were chosen to avoid toxicity, and because efflux monitoring is more sensitive than the MTT test to map mitochondrial effects. Mitochondrial respiration is the most efficient process for energy production, and dysfunction may lead to oxidative stress, organelle damage, and disturbed cell death. Mitochondrial dysfunction in Caco‐2 and HepG2 cells was studied by measuring oxygen consumption rate (OCR, a proxy for mitochondrial respiration) and extracellular acidification rate (ECAR, a proxy for glycolysis) followed by lactic acid secretion upon sequential treatment with oligomycin, FCCP and antimycin/rotenone. The results for Caco‐2 and HepG2 are shown in Figures 4 and 5.

Upon treatment of Caco‐2 cells with the five compounds, the total OCR can be ranked from high to low as follows: untreated > FM045 > FM038 > FM044 > Curcumin > FM050 (Figure 4A). Mitochondrial respiration was significantly impaired, and basal respiration, ATP production, and maximal respiration were considerably decreased compared to the untreated cells (*p* < 0.05, Figure 4B). The OCR of FM045, FM038, and FM044 significantly exceeded that of curcumin (*p* < 0.05, Figure 4B), demonstrating that curcumin was more performant in decreasing mitochondrial respiration compared to these analogs. FM050 had the highest capacity to inhibit both mitochondrial respiration and glycolytic activity (Figure 4A,C). Curcumin also decreased the glycolytic activity of Caco‐2 cells, but FM044, FM038, and especially FM045 treatment maintained glycolysis, thereby indicating that cells were still viable at the applied doses for the latter compounds. The energy map (Figure 4D) shows that, in contrast to Caco‐2 cells treated with FM044, curcumin, or FM050, the control cells and those treated with FM038 and FM045 use aerobic glycolysis and were more energetic. FM050 induced metabolic quiescence. Curcumin and especially FM044 shifted Caco‐2 metabolism from aerobic respiration towards anaerobic glycolysis (high ECAR, low OCR).

Results for HepG2 cells were not always following the same pattern as for Caco‐2 cells (Figure 5). The five compounds showed no significant effect on mitochondrial respiration compared to the control (*p* < 0.05, Figure 5B). The energy map revealed that, compared to the control, HepG2 cells treated with curcumin showed a metabolically quiescent phenotype, whereas cells treated with the other four analogs maintained a glycolytic profile (Figure 5D). Overall, the results suggest that curcumin and its analogs (especially FM050) decrease mitochondrial respiration and glycolysis in Caco‐2 cells, whereas such an inhibitory effect was not visible in HepG2 cells (*p* < 0.05).

## 4 Discussion

Structural modifications of curcumin significantly influence its bioactivity and selectivity, as observed in the current benzothiazepane analogs. Pyridine‐substituted derivatives (FM038, FM045, FM050) generally exhibited stronger anti‐proliferative effects against Caco‐2 cells compared to the furan‐substituted FM044, which was essentially non‐toxic. This pattern aligns with other studies on plant‐derived bioactive compounds, where heterocyclic moieties and core substitutions critically modulate cytotoxic potency (Aktepe et al. 2025). Similarly, the differential response between proliferating Caco‐2 and HepG2 cells underscores cell‐type‐specific mechanisms, consistent with reports on how environmental stressors and structural features alter physiological outcomes in plant and mammalian cell models (Günal‐Köroğlu et al. 2025). In our co‐culture system, intestinal metabolism further modulated the effects on HepG2 viability, highlighting the importance of gut‐liver axis interactions beyond direct cytotoxicity.

### 4.1 Structural Determinants of Anti‐Proliferative Activity in Cell Monocultures

The anti‐proliferative effect against Caco‐2 cells varied among curcumin and its analogs, according to their structural features. Curcumin's diketone and phenolic hydroxy groups may lead to increased intracellular reactive oxygen species and thus contribute to cytotoxicity (Nelson et al. 2017). However, the introduction of heterocyclic groups (pyridin‐3‐yl, pyridin‐2‐yl, or furan‐2‐yl) and methylation significantly altered bioactivity in our setup. By correlating MTT results (Table 1) with the structural characteristics of individual components (Figure 1), it could be concluded that the anti‐proliferative activity against Caco‐2 cells was governed by synergistic interactions between heterocyclic substituent identity, positional isomerism, and benzene ring methylation.

Pyridine derivatives universally outperformed the furan‐substituted analog (inactive FM044), demonstrating the critical role of nitrogen‐containing aromatic systems for anti‐proliferative activity. Compounds containing pyridine and furan moieties generally demonstrated promising cytotoxicity against cancer cells, which may arise from their high affinity for the active site of human topoisomerase IIβ, indicating their role as selective inhibitors of this enzyme (Kadi et al. 2023). The pyridine 3‐yl substitution (FM038) provided superior potency (IC_50_ ≈ 26 μM, comparable to curcumin) versus 2‐yl derivatives (FM045/FM050: IC_50_ > 33 μM), suggesting that the 3‐yl substitution probably led to optimal spatial alignment for target engagement and was therefore more effective at inhibiting proliferation in Caco‐2 cells than the 2‐yl derivatives.

The most significant structural difference between FM045 and FM050 was methylation of the benzothiazepane core in FM050 (Figure 1). This modification enhanced hydrophobicity, facilitating transmembrane permeability (Lu et al. 2006). Consequently, FM050 probably had superior cell membrane penetration compared to FM045, resulting in stronger anti‐proliferative activity against Caco‐2 cells.

In contrast to Caco‐2 cells, both curcumin and its four analogs showed no anti‐proliferative activity against HepG2 cells in a short‐term monoculture setup, thereby indicating that increased membrane permeability alone was probably not the only mode‐of‐action triggering cytotoxicity. However, it should be noted that the membranes of HepG2 and Caco‐2 cells may differ in lipid composition. For example, the higher cholesterol levels in HepG2 membranes may increase membrane rigidity while decreasing permeability (Raffy and Teissié 1999; Tsuji et al. 2021), which may further contribute to the lower uptake of lipophilic compounds. FM038, FM045 and FM050 (pyridinyl‐substituted analogs) showed negligible toxicity to HepG2 cells, possibly due to their reduced cellular uptake and/or metabolic inactivation mediated by their bulkier pyridine rings compared to furane (Zhang and Tsao 2016). This effect may be partially attributed to the higher intrinsic detoxification capacity of liver compared to intestinal cells, mediated by Phase II detoxifying enzymes and may reduce xenobiotic‐induced hepatocyte damage (Lee et al. 2016). It has also been reported that curcumin increased the detoxification capacity of hepatocytes by enhancing Nrf2‐ARE pathway‐mediated Phase II detoxifying enzymes and by mitigating oxidative damage, thereby recovering HepG2 cell viability (Garg et al. 2008). However, whether this effect of the analogs occurs through the same mechanism as curcumin—specifically by activating Phase II detoxification enzymes—requires further investigation.

A second explanation may be the higher cancerous nature of the HepG2 compared to the Caco‐2 cell line, as the latter is not able to develop tumours. Enhanced detoxification is typically driven by upregulation of multidrug resistance (MDR) proteins, which occurs when tumour cells develop resistance to a broad spectrum of structurally and functionally unrelated cytotoxic agents. This resistance stems from the overexpression of a shared efflux system utilized by multiple drugs. MDR transporters can export not only unmetabolized drugs and endogenous substrates but also Phase I and Phase II drug metabolites, leading to their designation as the Phase III detoxification system (Cole and Deeley 1998). Prominent MDR transporters include P‐glycoprotein (P‐gp) and multidrug resistance protein 1 and 2 (MRP1/MRP2) (Harris and Jeffery 2008). Whereas HepG2 cells express all three transporters, Caco‐2 cells lack MRP1. This may create a detoxification advantage of HepG2 over Caco‐2 cells (Keppler et al. 1997), which may explain the cell‐specific toxicity of curcumin and its analogs in our results. It also aligns with other studies showing that structural modifications may alter substrate specificity for metabolic enzymes, thereby influencing cell‐specific toxicity (Gupta et al. 2011). Yet, the structure–function relationships between curcumin and its analogs, their absorption and metabolism in Caco‐2 and HepG2 cells, as well as their effect on MDR transporter activity, require further investigation.

### 4.2 Metabolic Modulation in Co‐Culture Models

The intestine serves as the first barrier involved in the transport and metabolism of dietary bioactive components and determines the quantities and structure of components entering the systemic circulation and tissue/organ uptake and function. In this study, a co‐culture cell model was established to simulate the “gut‐liver” axis, aiming to investigate the effects of post‐intestinal metabolic products on hepatocyte viability. In this context, the differentiated Caco‐2 cells acted as an enterocyte model in contrast to the proliferating cell model in the previous experiments. Differentiated Caco‐2 cells largely lose their cancer‐like characteristics and develop mature enterocyte features, including brush borders with microvilli and altered enzymatic and transport systems (Stierum et al. 2003). Our results show that, unlike curcumin, treatment with the four analogs decreased the intestinal epithelial barrier integrity (Figure 2). In the literature, curcumin has been reported to enhance intestinal epithelial barrier integrity by upregulating tight junction protein expression and reducing intestinal epithelial cell apoptosis (Zhou et al. 2021). Whether these modes of action were affected by the four analogs needs further investigation. Yet, the overall TEER decrease was small, and the barrier was relatively well maintained upon longer‐term treatment with 7.5 and 15 μM concentrations (Vigh et al. 2021). We may therefore conclude that differentiated intestinal cells are more resistant to the cytotoxic effects of the analogs compared to the undifferentiated cells, and that the decreased TEER may be caused by altered cell morphology, (tight) junction expression, metabolism or decreased resistance to ROS, although this must be confirmed. Overall, we may conclude that the effects on the basal HepG2 cells were not the result of the diffusion of the compounds through gaps in the intestinal monolayer.

Literature reports that curcumin is transported through a Caco‐2 monolayer, although rather poorly with a *Papp* of 1.13 × 10^−6^ cm/s at 25 μM (Zheng et al. 2013), and this curcumin concentration is relatively high compared to those used in this study (7.5 and 15 μM). This implies that the concentration reaching the basal compartment may be relatively low, which is insufficient to cause cytotoxicity in HepG2 cells. In contrast, although the direct exposure of FM044, FM045, or FM050 to HepG2 cells did not result in acute toxicity (Table 1), a decreased HepG2 confluency was observed upon long‐term treatment with 15 μM apical exposure (*p* < 0.01, Figure 2). This may be explained in multiple ways. First, it is possible that the compounds were transported intact and caused effects in the longer term. Second, it may also be possible that FM044, FM045 and FM050 were degraded or metabolized to structures with altered solubility and membrane‐permeating capacities, uptake amount, or reactivity. Literature reports that drug‐metabolizing enzymes (DMEs) are highly upregulated upon Caco‐2 cell differentiation (Lnenickova et al. 2020) and may thus participate in the conversion of FM044, FM045, and FM050 into hepatotoxic metabolites. On the other hand, Caco‐2‐mediated metabolism (e.g., glucuronidation or sulfation) may decrease the activity of curcumin (Heger et al. 2014; Islam et al. 2024). A recent study of De Munck et al. (2026), in which LC–MS‐QTOF was used to identify metabolites generated from similar thiazepane‐based curcumin derivates, may further strengthen the hypothesis that all compounds are differently metabolized. The study demonstrates that compound AT164, corresponding to demethylated FM038, was less stable in presence of differentiated intestinal IPEC‐J2 cells compared to intestinal HCT116 cancer cell line. Hence, FM038 may have already been strongly metabolized to less toxic conversion products by the differentiated Caco‐2 cells in the coculture system, whereas it was toxic for the undifferentiated Caco‐2 in the viability experiments. In the study of De Munck et al. (2026), this stability effect was especially visible for the (only) tested pyridine 3‐yl substituted AT164, and less for the 4‐hydroxyphenyl substitutes. In addition, sulfation was observed for the latter compounds and curcumin, and not for AT164, which may explain the lower toxic effects of curcumin for the HepG2 cells in the basal compartment in our study. Interestingly, we also showed that the furanyl substituted FM044, with oxygen at the 2 position, showed a relatively similar cytotoxicity for the HepG2 cells in the coculture system as the pyridine‐2‐yl substituted FM045 and FM050. We may thus hypothesize that substitution at the 2‐position may result in transported metabolites with higher antiproliferative effects. However, further studies in which such metabolites are applied to the cells are needed to confirm this hypothesis. Finally, it is also possible that no curcuminoid metabolites have crossed the intestinal barrier, and that the effects are caused by cytokine‐driven crosstalk between the two cell types. Curcumin has been shown to alter (pro‐)inflammatory stimuli such as TNF‐α and IL‐1 (Gorabi et al. 2021). These cytokines have a profound effect on lipid, sugar and amino acid metabolism in liver cells (Kim et al. 2007; Xu et al. 2025) and reduce cell viability (Gomaa et al. 2024). Which of these effects have occurred in our model needs to be further explored by for instance analytical metabolite profiling and molecular determination of the mode‐of‐actions.

### 4.3 Mitochondrial Dysfunction as a Mechanistic Driver

Since mitochondrial dysfunction has been reported upon curcumin treatment, and the resulting ROS imbalance may lead to altered barrier effects, pro‐inflammatory cytokine release and cell growth, we further investigated this using efflux analysis (OCR and ECAR) upon extra mitochondrial stressors. For Caco‐2 cells, curcumin and FM050 strongly inhibited both mitochondrial respiration and glycolysis (Figure 4), which may directly point to cytotoxicity as confirmed by MTT in Caco‐2 cells (Figure S1; Table 1). Curcumin has been shown to accumulate in the mitochondria, and, depending on the dose, the effect on mitochondrial function may be dual. On the one hand, short‐term high‐dose administration may result in mitochondrial damage and cytochrome c release depending on the cell type, which may be linked to the ROS‐inducing and protonophore capacities of high doses (Tabassum and Parvez 2021). On the other hand, low‐dose long‐term addition may result in increased mitochondrial fusion activity, reduced fission machinery, and increased biogenesis, as well as increased PGC‐1α expression, improved mitochondrial potential and ATP‐production (Tabassum and Parvez 2021). At high doses, it has been reported that curcumin cytotoxicity is triggered by more events than mitochondrial dysfunction alone (Tabassum and Parvez 2021; Joshi et al. 2021). In addition to modulating numerous cell‐regulating pathways, curcumin has been shown to inhibit key glycolytic enzymes such as hexokinase II and lactate dehydrogenase, as well as glucose transporters (GLUT1/4), leading to reduced glucose uptake and lactic acid production (Gueguinou et al. 2022; Rukoyatkina et al. 2021; Wang et al. 2015).

Our bioenergetic profiles suggest that FM050 may work in a similar fashion in growing Caco‐2 monolayers, despite its higher similarity to the other analogs. On the one hand, this may be partially explained by the higher affinity for cell organelle (mitochondrial) membranes of the core methylated group. Cellular methylation of quercetin to products that accumulate in the mitochondria of Caco‐2 cells has been demonstrated before (Vissenaekens et al. 2021). In addition, colocalization of a very similar methylated curcumin thiazepane analog, but with a 4‐hydroxyphenyl group at the position of the pyridine‐2‐yl, upon addition to or production from the non‐methyl derivative by Caco‐2 cells, was suggested by De Munck et al. (2026), thereby suggesting a crucial role of the core methylated group for mitochondrial accumulation. Although the effect of methylation on mitochondrial effects is not well explored, it is known that methylated proteins in the mitochondria are fundamental constituents of the bioenergetics machinery, such as respiratory Complex 1, citrate synthase, and ATP synthase (Malecki et al. 2022). Yet, the absence of such an effect for the methylated compound FM038 may suggest that the position of the N in the pyridinyl‐group may be crucial for the mitochondrial effect. Indeed, the pyridine‐3‐yl group, and not the pyridin‐2‐yl group, is structurally similar to pyrimidine‐containing nucleotides, which play an essential role in maintaining mitochondrial pyruvate oxidation (Sahu et al. 2024).

Yet in the coculture experiments, TEER and HepG2 cell viability were more affected by FM050 than by curcumin (Figure 2). This feeds the hypothesis that modified bioavailability, detoxification, and stability mechanisms have caused this effect. The remaining three compounds (FM038, FM044, and FM045) decreased OCR while increasing ECAR in Caco‐2 cells, suggesting a metabolic shift toward glycolysis and thus a more moderate effect, which can be induced by cellular reprogramming (de la Maza et al. 2017).

Curcumin's suppression of respiration in both Caco‐2 and HepG2 cells (Figures 4 and 5) correlated with its known role as a mitochondrial inhibitor (Bordoloi et al. 2016). These four analogs effectively changed the energy metabolism pattern in Caco‐2 cells, whereas they barely affected HepG2 cells (*p* < 0.05, Figures 4 and 5), confirming their selective effect on the two cell lines. According to MTT results (Table 1), FM038 exhibited toxicity like curcumin but higher than FM050 in Caco‐2 cells, while FM044 showed no toxicity. In contrast, efflux analysis revealed that FM044 significantly decreased aerobic glycolysis and reduced ATP production. Furthermore, FM050‐treated Caco‐2 cells exhibited a metabolically quiescent phenotype (*p* < 0.05, Figure 4), indicating reduced metabolic activity and compromised energy supply. Thus, it could be inferred that the toxic effect was more apparent in the more sensitive efflux analysis than in the MTT assay, and FM050 may exert cytotoxic effects by targeting the mitochondria of Caco‐2 cells. As for HepG2 cells, as previously mentioned, we propose that hepatocytes mitigate mitochondrial toxicity via intrinsic detoxification mechanisms and altered uptake. These analogs showed almost no influence on OCR, and FM044 and FM045 even increased ECAR (Figure 5C). The enhanced glycolysis may promote cell survival under metabolic stress (Liberti and Locasale 2016), explaining their non‐cytotoxicity on HepG2 cells. Overall, we may conclude that benzothiazepane derivatives improve the selectivity of the curcuminoid beyond mitochondrial dysfunction.

## 5 Conclusion

**FIGURE 6:**
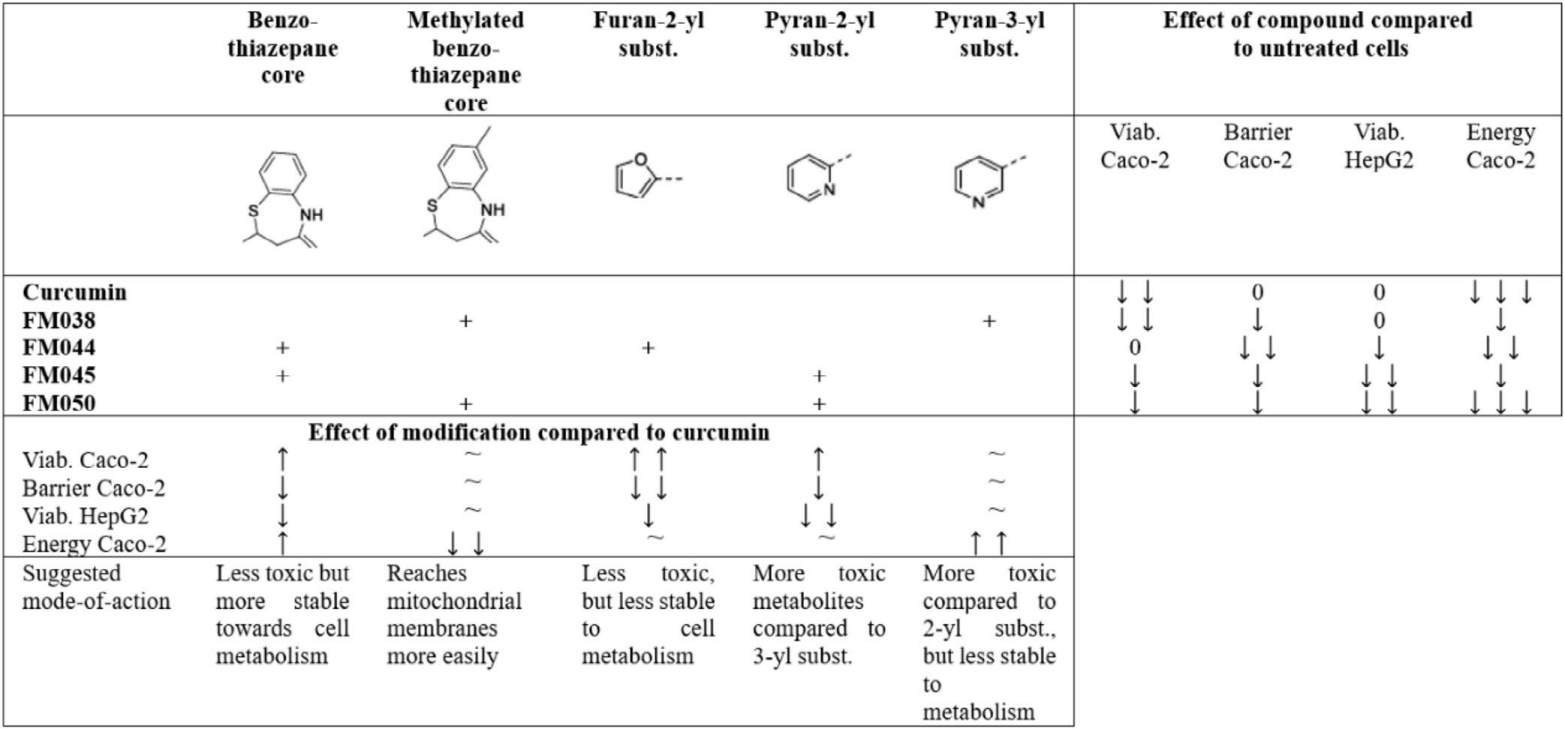
Schematic overview of the major structure–activity (SAR) based on core modifications (benzothiazepane, either or not methylated) and side chain substitutions (furan‐2‐yl, pyran‐2‐yl or pyran‐3‐yl) compared to the original curcumin molecule. +: Modification is present, ~: Unclear effect, **↑**: Increased effect,**↓**: Decreased effect, 0: No effect.

This study demonstrates that benzothiazepane‐based structural modifications of curcumin critically modulate its selective cytotoxicity, barrier and mitochondrial effects in Caco‐2 and HepG2 cells in a cell‐specific and dose‐dependent way (Figure 6). Compared to curcumin, both FM045 and FM050—which feature pyridin‐2‐yl substituents but differ in methyl group presence on the benzothiazepane core (FM050: methylated)—decreased IC_50_ values in Caco‐2 cells, whereas FM044, bearing furan‐2‐yl substituents, was non‐toxic. In contrast, FM038, with a methylated benzothiazepane core and pyridin‐3‐yl groups, showed Caco‐2 anti‐proliferative activity comparable to curcumin. Interestingly, the direct exposure of these compounds to proliferating HepG2 cells did not induce toxic effects, indicating a cell‐specific anti‐proliferative mode‐of‐action. Nevertheless, following passage through differentiated Caco‐2 cells (co‐culture model), the potentially altered structures of FM044, FM045, and FM050 (15 μM) significantly reduced HepG2 cell growth. This suggests that the metabolic processes in differentiated Caco‐2 cells may potentiate the anti‐proliferative activity in HepG2 cells, which may be linked to differences in compound stability, uptake and function, although effects mediated by cellular crosstalk cannot be excluded. In contrast to the other analogs, bioenergetic efflux analysis showed that FM050 induced mitochondrial dysfunction and deficient glycolysis in both Caco‐2 and HepG2 cells, comparable to curcumin. This highlights the key role of the methyl group on the benzothiazepane core, as such effects were not observed with the methyl‐free FM045 structure. Other compounds compensated for the loss in mitochondrial function with increased glycolysis, indicating a more moderate effect on cellular energy production pathways. Overall, the results suggest that for these compounds, the interplay between stability, detoxification, altered uptake, diffusion, and indirect cell–cell communication will direct the final effect in the body.

These findings highlight the potential of targeted structural optimization to improve cancer‐specific bioactivity and the essential role of colonic cell metabolism in mediating anti‐hepatocellular carcinoma efficacy. However, limitations are that these findings were only demonstrated by in vitro models from a cancer origin, and thus lack validation with non‐cancer cells, in vivo models, and detailed metabolite characterization. Future work should use analytical and molecular tools to further explore the mode‐of‐action at the cellular level, establish pharmacokinetics in vivo, elucidate metabolic pathways, and refine substituent design to enhance selectivity while minimizing off‐target effects, thereby advancing curcumin‐based chemo‐preventive agents.

## Author Contributions

**Fuhua Li:** conceptualization, data curation, investigation, formal analysis, writing – original draft. **Julie De Munck:** conceptualization, data curation, investigation, formal analysis, visualization, writing – review and editing, funding acquisition. **Felien Morlion:** resources, writing – review and editing. **Katarina Magdalenic:** resources. **Andreja Rajkovic:** funding acquisition. **Matthias D'hooghe:** funding acquisition, writing – review and editing. **John Van Camp:** conceptualization, supervision, funding acquisition, writing – review and editing. **Charlotte Grootaert:** conceptualization, data curation, investigation, formal analysis, supervision, funding acquisition, writing – review and editing.

## Funding

This work was supported by China Scholarship Council (202006995003), Bijzonder Onderzoeksfonds UGent (BOF20/IOP/006, BOF.BAS.2020.0035.01), Fonds Wetenschappelijk Onderzoek (FWO‐WEAVE G000123N, 1S05525N).

## Ethics Statement

This study was performed in line with the principles of the Declaration of Helsinki. Approval for the collection and use of commercially available human cell lines was granted by the Ethics Committee of Ghent University (2019/0914).

## Consent

The authors have nothing to report.

## Conflicts of Interest

The authors declare no conflicts of interest.

## Supporting information


**Figure S1:** Cell viability (%) of proliferating Caco‐2 cells treated with curcumin or analogs (0–40 μM) for 24 h in a monolayer setup, assessed by MTT conversion. Distinct letters denote significant differences (*p* < 0.05).
**Figure S2:** Cell viability (%) of proliferating HepG2 cells treated with curcumin or analogs (0–40 μM) for 24 h in a monolayer setup, assessed by MTT conversion.
**Figure S3:** TEER values (Ω/well) of differentiated Caco‐2 cells in the apical compartment of a co‐culture set‐up, exposed to curcumin, or analogs (7.5 and 15 μM) for 0‐96 h.
**Figure S4:** Confluence percentage of proliferating HepG2 Cells in the basal compartment of a co‐culture set‐up, after apical exposure to curcumin or analogs (7.5–15 μM) for 0–96 h.

## Data Availability

Relevant datasets generated during and/or analysed during the current study are visualized in Supporting Information S1, and raw data are available from the corresponding author upon request.
