# Supplementary material for: Benzothiazepane‐Based Curcumin Analogs Show Selective Effects on Respiration, Permeability, and Growth of Gut‐Liver (Co‐Culture) Cell Models
Source: Chem Biol Drug Des. 2026 Sep 21;108(3):e70406. doi: 10.1111/cbdd.70406 (PMC13594503; doi:10.1111/cbdd.70406)
Supplement: Supplementary file 1 [file CBDD-108-e70406-s001.docx]

**Supplementary data**

**Figure legends**

**Supplementary Fig1** Cell viability (%) of proliferating Caco-2 cells treated with curcumin or analogues (0-40 µM) for 24h in a monolayer setup, assessed by MTT conversion. Distinct letters denote significant differences (p<0.05)

**Supplementary Fig2** Cell viability (%) of proliferating HepG2 cells treated with curcumin or analogues (0-40 µM) for 24h in a monolayer setup, assessed by MTT conversion

**Supplementary Fig3** TEER values (Ω/well) of differentiated Caco-2 cells in the apical compartment of a co-culture set-up, exposed to curcumin or analogues (7.5 and 15 µM) for 0-96h

**Supplementary Fig4** Confluence percentage of proliferating HepG2 Cells in the basal compartment of a co-culture set-up, after apical exposure to curcumin or analogues (7.5-15 µM) for 0–96h

**Supplementary Fig1**

**Supplementary Fig2**

**Supplementary Fig3**

**Supplementary Fig4**
